# Strong effect of Anti-COVID-19 vaccination on the peak titer of antibodies against novel coronavirus: factors affecting humoral immunity in Georgian plasma donors

**DOI:** 10.1186/s13104-026-07839-x

**Published:** 2026-05-11

**Authors:** Tamar Azikuri, George Kamkamidze, Maia Butsashvili, Nikoloz Pruidze, Natia Kvaratskhelia

**Affiliations:** University of Georgia, 77a, M. Kostava str., Tbilisi, 0171 Georgia

**Keywords:** COVID-19, SARS-cov-2, Vaccination, Humoral immunity, Antibody response, ABO blood group, Rhesus factor, Hybrid immunity, Plasma donors, CAUCASUS region

## Abstract

**Objective:**

Humoral responses to COVID-19 vaccination vary across populations, and evidence on the role of ABO and the Rhesus factor remains inconsistent. This prospective cohort study examined peak antibody responses among 175 voluntary plasma donors in Georgia during 2022–2023, a setting where the prevalence of Rhesus negativity is relatively high and no prior data have been reported.

**Results:**

Using quantitative anti-spike IgG ELISA, we measured peak antibody titers over a six-month period following vaccination and/or natural infection. Vaccination emerged as the strongest predictor of high antibody response (≥ 1000 BAU/mL), with 81.9% of vaccinated versus 21.0% of unvaccinated participants achieving protective levels (OR = 16.88, *p* < 0.001). Rhesus-positive individuals were significantly more likely to achieve protective titers than Rhesus-negative (57.7% vs. 30.8%, *p* = 0.011), while ABO blood group showed no association with antibody response (*p* = 0.374). Hybrid immunity provided optimal protection, with 92.5% of participants achieving elevated titers. These findings highlight vaccination and hybrid immunity as central determinants of humoral response, while identifying the Rhesus factor as an additional contributor. This study represents the first dataset of its kind from the Caucasus region, providing distinct regional evidence with implications for global immunological research.

## Introduction

COVID-19 vaccination programs have effectively reduced severe disease and mortality worldwide [1, 2]. However, individual differences in immune response remain incompletely understood. Previous studies have reported mixed findings on the association between ABO blood group and SARS-CoV-2, with results varying by population and study design [3]. Understanding these determinants is essential for optimizing vaccination strategies in diverse populations.

Blood group antigens have been considered as potential modifiers of COVID-19 susceptibility and immunity. Some studies reported an association between blood group A and increased SARS-CoV-2 infection risk, whereas other reports did not replicate this finding; overall, the evidence remains mixed [3–5] The Rhesus factor has been examined in several studies with mixed findings: a few reports suggest associations with infection risk or antibody levels [3, 6], but overall evidence is inconsistent and few studies have specifically evaluated its effect on humoral responses after vaccination.

Hybrid immunity, resulting from vaccination combined with prior infection, has emerged as a key determinant of robust and durable antibody responses [7–12]. While this effect is well documented in Western and Asian populations, evidence is scarce from regions with distinct genetic and epidemiological profiles.

Georgia, located in the Caucasus, represents such a setting. The country experienced substantial SARS-CoV-2 transmission with comparatively modest vaccination uptake. The prevalence of Rhesus negativity in Georgia is higher than in many neighboring countries, making this population particularly relevant for studying blood group associations.

This study therefore aimed to assess peak antibody titers following vaccination and/or infection in Georgian plasma donors and to evaluate demographic, clinical, and genetic factors—including ABO blood group and the Rhesus factor—that may influence humoral immunity.

## Methods

### Study design and participants

We conducted a prospective cohort study among 175 healthy adults aged 18–65 years with documented vaccination and/or SARS-CoV-2 infection at least two weeks prior to enrollment. The study was carried out in Georgia between January 2022 and December 2023. Participants provided monthly blood samples for up to six months (≥ 4 per donor), enabling longitudinal monitoring of antibody dynamics and determination of each participant’s peak response.

### Antibody measurements and standardization

Anti-SARS-CoV-2 spike (RBD) IgG was measured using the Anti-SARS-CoV-2 S1-RBD IgG AccuBind^®^ ELISA (Monobind Inc., Product Code 12525-300) on EDTA plasma. Deidentified plasma samples were diluted 1 in the supplied buffered diluent and 100 µL of diluted sample and controls (positive, negative and kit Cut-Off Control) were added to microwells coated with recombinant S1-RBD antigen. Plates were incubated for 30 min at room temperature (20–27 °C), washed three times with the supplied wash buffer, then 100 µL anti-human IgG-HRP conjugate was added and incubated for 30 min. After a second triple wash, 100 µL tetramethylbenzidine (TMB) substrate was added for 15 min; the reaction was stopped with 50 µL 0.5 M H2SO4 and absorbance read at 450 nm (reference 620–630 nm). Results were calculated as Relative Values (RV) versus the kit Cut-Off Control per the manufacturer’s instructions; RV > 10 was interpreted as positive. The kit insert provides conversion to WHO-traceable units (RV × 11 = IU/mL, valid up to 25 RV) and we report results as BAU/mL after conversion. Each sample was tested in duplicate and the mean value used. For each donor, peak antibody titer was defined as the highest IgG concentration during follow-up; a threshold of ≥ 1000 BAU/mL was used to define a high (putatively protective) peak titer, in line with published correlates of protection. (Khoury et al. [2], Earle et al. [13]. Where a numeric diluted result was produced by the laboratory, that value (adjusted by dilution) was used; records recorded at the dataset cap (> 40,000 BAU/mL) with no higher numeric result were set to 40,000 BAU/mL for the primary analyses.

### Data collection

Baseline data included age, sex, vaccination history, and prior infection status. Vaccination records specified dose number and vaccine type (Pfizer-BioNTech, AstraZeneca, Sinovac, or Sinopharm). Prior SARS-CoV-2 infection was confirmed by polymerase chain reaction (PCR) when available or by clinical diagnosis. ABO blood group and the Rhesus factor were determined by standard hemagglutination methods. Participants were categorized as Rhesus-positive or Rhesus-negative.

### Sample size

The minimum required sample was estimated with G*Power. Assuming an effect size of W = 0.3, α = 0.05, and power = 0.80, 172 participants were needed. The final study included 175 donors.

### Statistical analysis

Descriptive statistics summarized participant characteristics. Categorical variables were expressed as frequencies and percentages; continuous variables as mean ± standard deviation (SD). Associations between categorical variables were assessed with Chi-square or Fisher’s exact test. Logistic regression was used to calculate odds ratios (ORs) with 95% confidence intervals (CIs) for predictors of protective antibody response (≥ 1000 BAU/mL). Independent variables included vaccination status, prior infection, ABO blood group, Rhesus factor, age, and sex. Significance was set at *p* < 0.05. Analyses were performed in SPSS v26.0 (IBM Corp., Armonk, NY, USA).

### Ethics

This prospective cohort study was conducted in compliance with the Declaration of Helsinki. Approval was obtained from the University of Georgia Institutional Review Board (reference number: 11-24768). All participants provided written informed consent prior to enrollment.

## Results

### Population characteristics

The study included 175 participants: 133 males (76.0%) and 42 females (24.0%), with a mean age of 32.4 years (SD = 9.8). Vaccination coverage was 53.7% (94/175), while 46.3% (81/175) were unvaccinated. Distribution of ABO groups was: O, 45.7%; A, 32.0%; B, 15.4%; and AB, 6.9%. For the Rhesus factor, 85.1% were Rhesus-positive and 14.9% Rhesus-negative (Table 1).

**Table 1:** Baseline characteristics of study population (*n* = 175)

Characteristic	*n*	% / Mean ± SD
Age (years)	–	32.4 ± 9.8
Sex		
Male	133	76.0
Female	42	24.0
Vaccination status		
Vaccinated	94	53.7
Unvaccinated	81	46.3
ABO group		
O	80	45.7
A	56	32.0
B	27	15.4
AB	12	6.9
Rhesus factor		
Positive	149	85.1
Negative	26	14.9

Values are presented as mean ± standard deviation (SD) or number (%)

### Effect of vaccination

Among Rh negative individuals 50% were vaccinated vs. 50% not vaccinated, while in Rh positives 45.6% were vaccinated vs. 54.4% not vaccinated. This difference was not statistically significant. (*p* = 0.68).

**Fig. 1:**
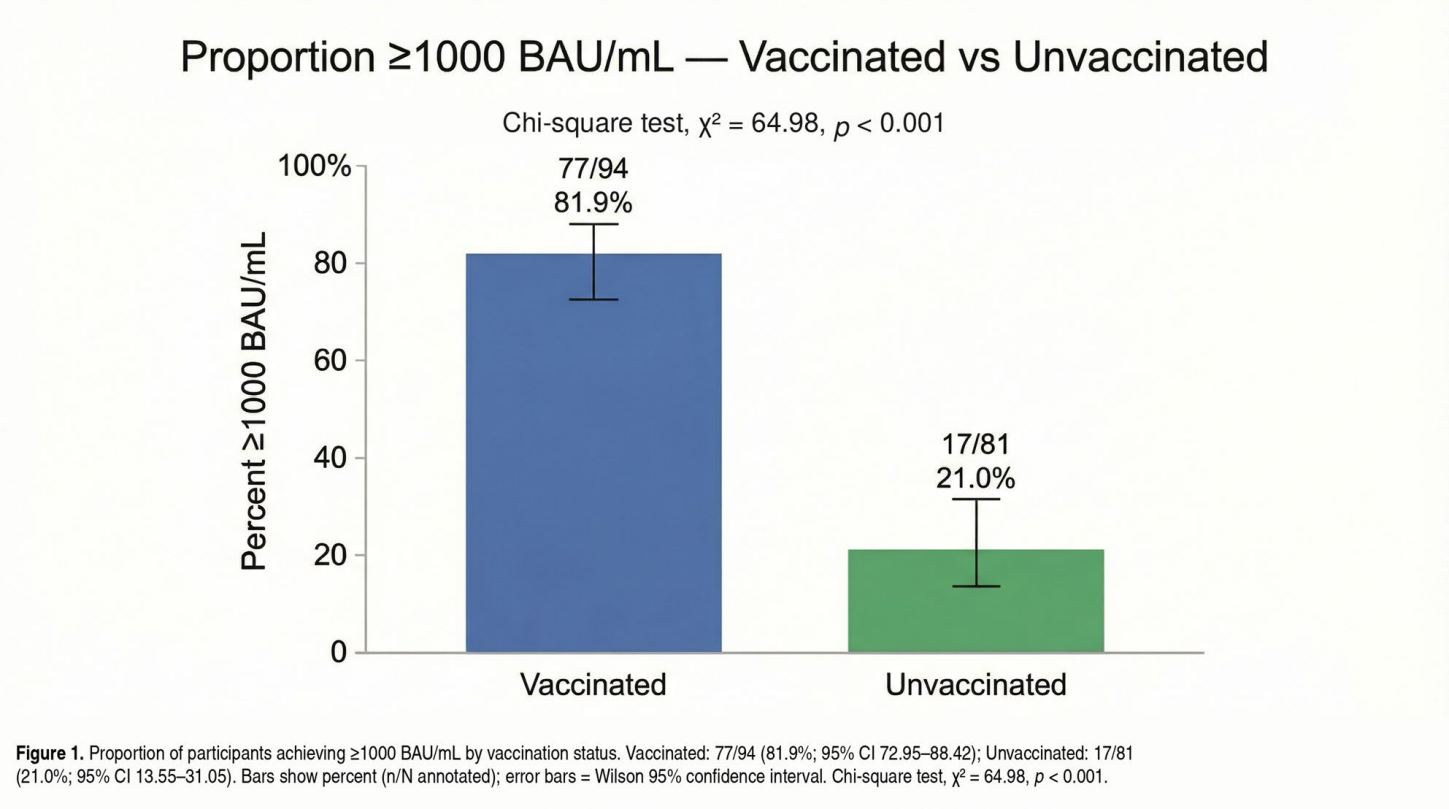
Antibody protection by vaccination status. Proportion of participants achieving protective antibody titers (≥1000 BAU/mL) according to vaccination status. Among vaccinated individuals, 81.9% achieved protective levels compared with 21.0% of unvaccinated. OR = 16.88 (95% CI: 8.54–33.37); χ² = 64.98; *p* < 0.001. Error bars indicate 95% CIs

Vaccination was the strongest predictor of protective antibody response. Among vaccinated participants, 81.9% (77/94) achieved titers ≥ 1000 BAU/mL compared with 21.0% (17/81) of unvaccinated participants (χ² = 64.98, *p* < 0.001) (Fig. 1). This corresponded to a 17-fold higher odds of achieving protective levels (OR = 16.88, 95% CI: 8.54–33.37), consistent with international evidence [2, 9, 10, 13].

### Genetic and demographic factors

ABO blood group showed no significant association with antibody response (χ² = 3.12, *p* = 0.374). Response rates were: O, 52.5%; A, 57.1%; B, 59.3%; AB, 33.3%. By contrast, the Rhesus factor significantly influenced antibody response: 57.7% (86/149) of Rhesus-positive participants achieved protective titers compared with 30.8% (8/26) of Rhesus-negative participants (χ² = 6.47, *p* = 0.011). Neither sex (females 61.9% [26/42] vs. males 51.1% [68/133], *p* = 0.222) nor age (*p* = 0.312) modified vaccine effectiveness. We report vaccination counts by Rhesus status: among Rhesus-positive donors 83/149 (55.7%) were vaccinated and 66/149 (44.3%) unvaccinated; among Rhesus-negative donors 11/26 (42.3%) were vaccinated and 15/26 (57.7%) unvaccinated. Stratified analyses by vaccination status indicate the observed Rhesus association with protective titers was not explained solely by differences in vaccination.

### Disease severity and immunity groups

Among unvaccinated participants, only 10.0% (4/40) with non-hospitalized COVID-19 achieved protective titers, compared with 90.9% (10/11) of previously hospitalized cases (Fisher’s exact *p* < 0.001). Among vaccinated participants, antibody levels did not differ by severity of prior disease (81.4% non-hospitalized vs. 87.5% hospitalized, *p* = 0.668).

**Fig. 2:**
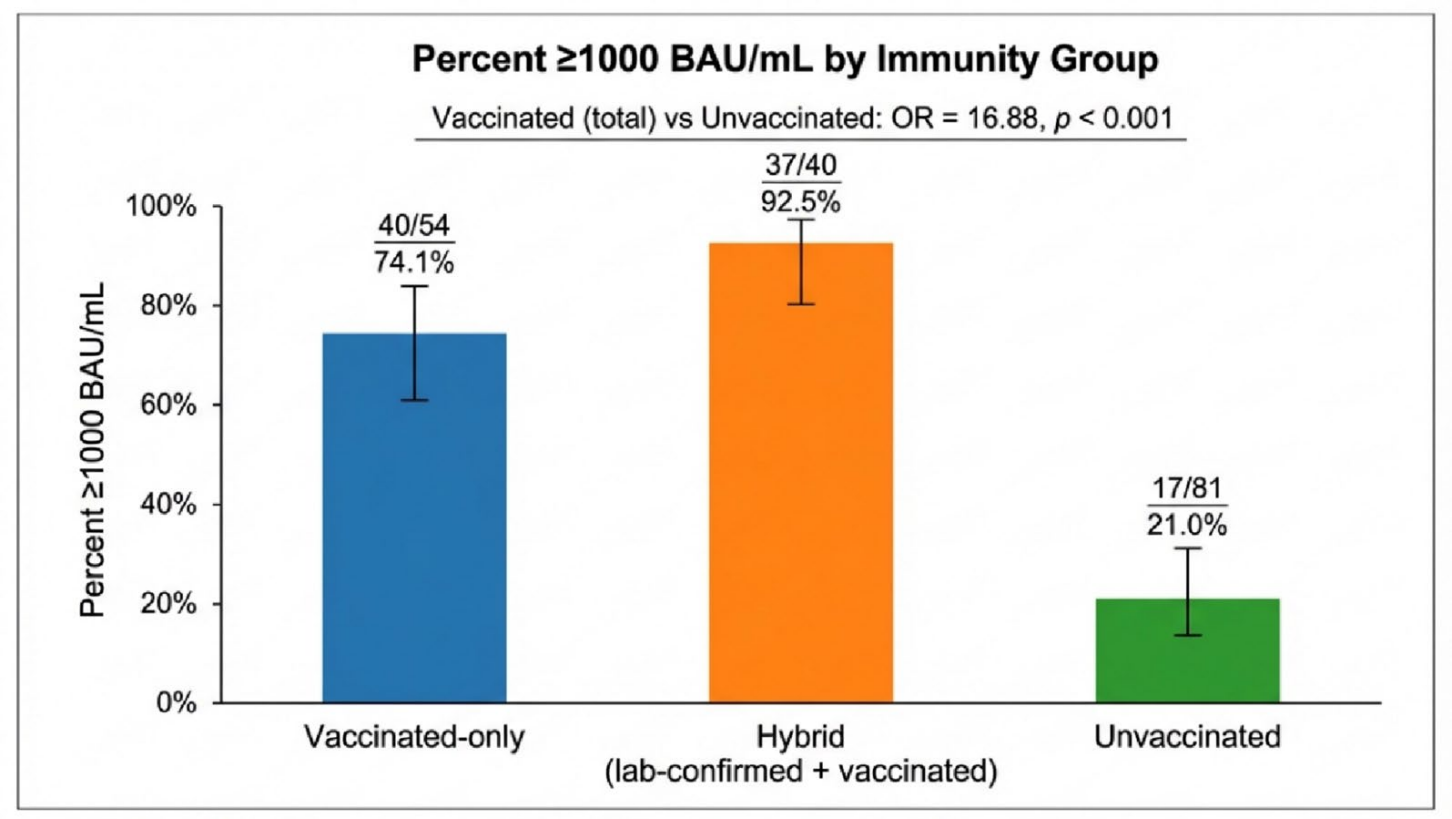
Antibody protection by immunity type. Protective antibody responses (≥1000 BAU/mL) by immunity group: unvaccinated (21.0%), vaccinated only (74.1%), and hybrid immunity (92.5%). Hybrid immunity conferred the highest protection, exceeding vaccination alone (*p* < 0.001). Error bars indicate 95% CIs

**Fig. 3:**
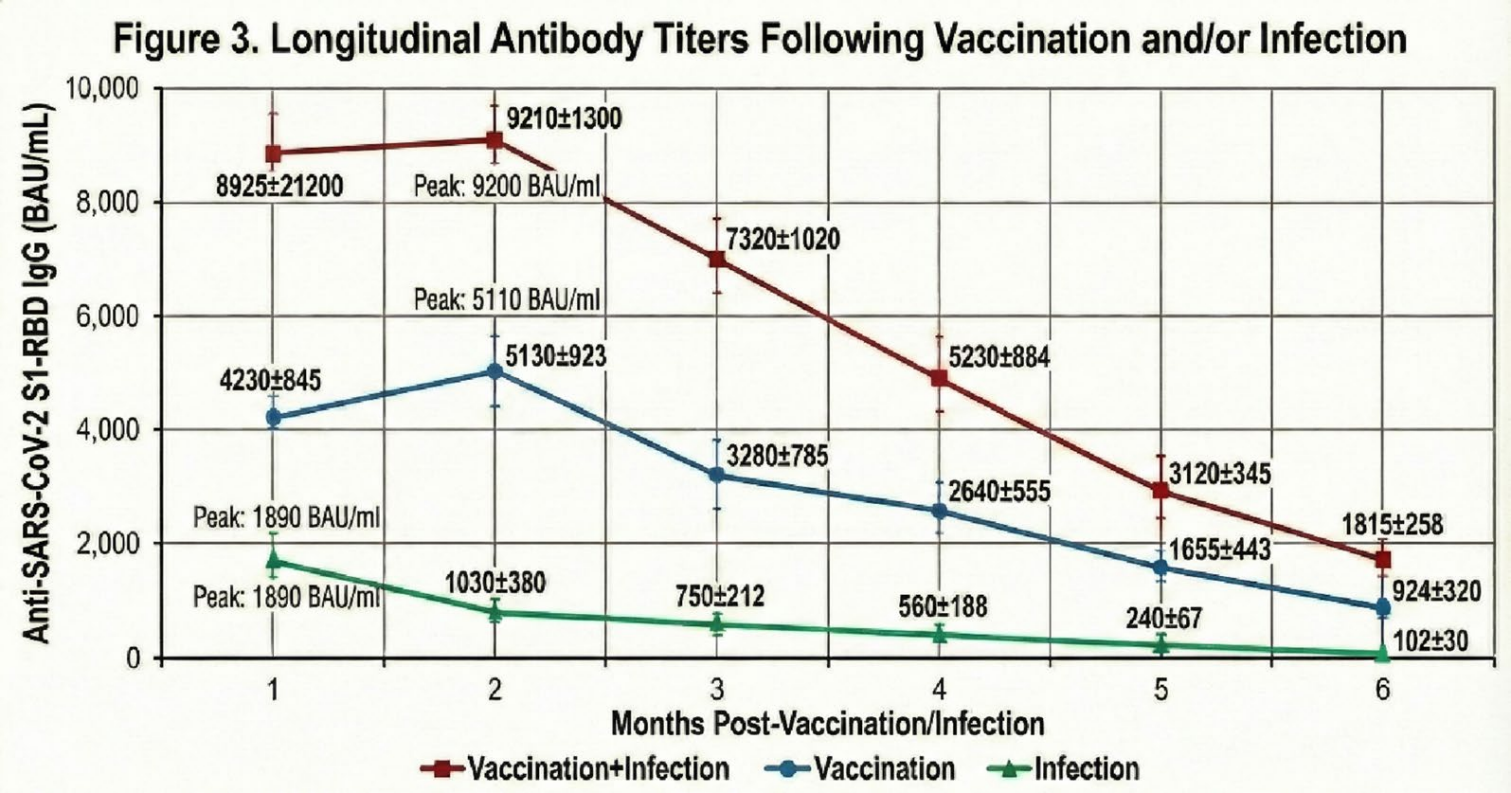
Longitudinal kinetics of anti-SARS-CoV-2 S1-RBD IgG and peak humoral response. Mean anti-spike IgG concentrations (BAU/mL) measured at monthly intervals over a six-month follow-up period. To ensure international comparability and adherence to WHO standards, raw laboratory values were converted to Binding Antibody Units (BAU/mL) using the manufacturer’s standardized conversion factor (RV × 11 = BAU/mL). The peak titer for each participant represents the maximum recorded concentration across the longitudinal monitoring period to account for individual variability in immune kinetics. Curves represent the mean titers for the three immunity cohorts (unvaccinated, vaccinated-only, and hybrid immunity), with error bars indicating the 95% confidence intervals (CIs). The horizontal dashed line represents the protective threshold of ≥1000 BAU/mL

Hybrid immunity provided the highest protection: 92.5% of these individuals achieved protective titers, significantly exceeding vaccination alone (*p* < 0.001). (Figures 2 and 3). These findings align with global reports of hybrid immunity as the most robust form of protection [7–11].

## Discussion

In this prospective cohort of Georgian plasma donors, vaccination and hybrid immunity were the strongest determinants of protective antibody response, while the Rhesus factor emerged as an additional influence. ABO blood group, age, and sex showed no effect. To our knowledge, this is the first study from the Caucasus to evaluate humoral immunity after COVID-19 vaccination and infection, contributing data from a population with relatively high Rhesus negativity.

### Vaccination as the dominant determinant

Vaccinated participants were nearly 17 times more likely to achieve titers ≥ 1000 BAU/mL than unvaccinated individuals, confirming the central role of vaccination [1, 2, 9, 10, 13]. This threshold, based on WHO standards, has been associated with reduced infection risk [2, 13]. Protection was consistent across age and sex, supporting findings from other cohorts [14–16].

### Role of the rhesus factor

Rhesus-positive donors were significantly more likely to achieve protective titers than Rhesus-negative. While ABO has been widely studied with mixed results [4, 17], evidence on Rhesus is limited [3, 5, 6]. Our results suggest a potential immunological role, possibly through genetic linkage or antigen-driven immune modulation [3]. This observation warrants confirmation in larger, multi-ethnic cohorts.

### Lack of ABO association

We observed no significant differences between ABO groups, consistent with recent meta-analyses reporting minimal impact on SARS-CoV-2 infection or immunity [4, 17].

### Hybrid immunity as optimal protection

Hybrid immunity provided the strongest protection, with 92.5% achieving protective titers, consistent with international findings [7–12, 18, 19]. This underscores the benefit of vaccinating even previously infected individuals, particularly in Georgia where infection prevalence was high and vaccine uptake modest.

### Influence of disease severity

Among unvaccinated participants, severe infection produced stronger antibody responses than mild infection, but vaccination overcame this variability, confirming the reliability of vaccine-induced protection [9, 16].

## Limitations

This study has several limitations. First, the sample consisted exclusively of healthy adult plasma donors, which may limit the generalizability of the findings to older populations or individuals with comorbidities. Second, antibody levels were measured only by quantitative anti-spike IgG ELISA; cellular immune responses and neutralizing activity were not assessed, and therefore the results reflect only one aspect of the immune response. Third, vaccine type and number of doses were documented but not analyzed separately due to limited subgroup sizes, which may have obscured differences between vaccine platforms. Fourth, infection status was based partly on self-report and clinical diagnosis, introducing the possibility of misclassification for participants without PCR confirmation. Finally, the study was conducted in a single country, and the influence of the Rhesus factor on antibody response should be validated in larger, multi-center cohorts.

## Conclusions

This prospective cohort study of 175 plasma donors in Georgia identified vaccination as the strongest predictor of protective antibody titers, with vaccinated individuals nearly 17 times more likely to achieve the ≥ 1000 BAU/mL threshold than unvaccinated participants. Hybrid immunity, combining natural infection with vaccination, conferred the highest protection, with more than 92% achieving protective levels. These results align with international evidence and emphasize the added value of vaccination, even among previously infected individuals.

A novel finding was the association between the Rhesus factor and antibody response. Rhesus-positive individuals were significantly more likely to achieve protective titers than Rhesus-negative participants. While mechanisms remain unclear, this observation adds to limited evidence that red cell antigens may influence immune responses to SARS-CoV-2. Confirmation in larger, multi-ethnic studies is needed.

These findings carry both scientific and practical implications. Scientifically, they highlight the need to integrate host genetic factors such as the Rhesus factor into studies of vaccine effectiveness. Practically, they suggest that Rhesus-negative individuals may represent a subgroup less likely to reach protective immunity, raising the possibility of tailored vaccination strategies.

Taken together, the results support vaccination as the cornerstone of COVID-19 control and demonstrate the additive benefit of hybrid immunity. The observed Rhesus effect introduces a new direction for research on host determinants of immune variability and may help refine approaches to vaccination.

## Data Availability

The datasets generated and analyzed during the current study are available from the corresponding author upon reasonable request.

## Abbreviations

BAU: Binding antibody units
CI: Confidence interval
COVID-19: Coronavirus disease 2019
ELISA: Enzyme-linked immunosorbent assay
IgG: Immunoglobulin G
OR: Odds ratio
PCR: Polymerase chain reaction
SARS-CoV-2: Severe acute respiratory syndrome coronavirus 2
SD: Standard deviation
